# Commentary: The Moral Obligation to Prioritize Research Into Deep Brain Stimulation Over Brain Lesioning Procedures for Severe Enduring Anorexia Nervosa

**DOI:** 10.3389/fpsyt.2019.00634

**Published:** 2019-09-03

**Authors:** Chencheng Zhang, Zhengyu Lin, Wenying Xu, Wei Liu, Dianyou Li, Guozhen Lin, Bomin Sun

**Affiliations:** ^1^Department of Functional Neurosurgery, Ruijin Hospital, Shanghai Jiao Tong University School of Medicine, Shanghai, China; ^2^INSERM U1266, Institute of Psychiatry and Neuroscience of Paris (IPNP), Paris, France; ^3^Department of Psychiatry, Ruijin Hospital, Shanghai Jiao Tong University School of Medicine, Shanghai, China

**Keywords:** deep brain stimulation, anorexia nervosa, accumbens, subgenual anterior cingulate, capsulotomy

We appreciate the comment of Pugh et al. on our research report concerning capsulotomy for refractory anorexia nervosa (AN) (1). However, we disagree with their view that “lesioning procedures in severe and enduring AN are unethical at this stage of knowledge and seriously problematic for this patient group” (2). Instead, as subsequently stated by Zrinzo et al. (3), we think that “the real moral obligation is to pursue every possible avenue.” While the latter authors offer a thoughtful rebuttal to the main criticism made by Pugh et al., we provide here some additional information on the clinical protocol utilized in our study. As acknowledged in the research report (1), our clinical protocol partly differed from the protocols most commonly used in the field, which raised some more specific issues of concern by Pugh et al.

Our study differed with respect to the eligibility criteria typically used in deep brain stimulation (DBS) treatment studies of AN. (4) We included only patients who suffered from AN for more than 3 years and who did not respond to conventional pharmacotherapy and psychotherapy. The psychotherapy previously given to the patients lasted for no longer than 3 months because the use of short-term psychotherapy was common clinical practice at that time in China. By comparison, as underlined by Pugh et al., DBS treatment studies of AN have usually included only patients with an illness duration of at least 7 years. To be eligible for DBS in these studies, the patients were also required to have a history of insufficient clinical response to conventional treatments for AN, including a psychological treatment lasting for at least 6–12 months. Yet, our definition of illness duration is not without precedent. The number of years of illness reported in the literature ranges from 3 to 10, with a mode of 7 (5, 6). Moreover, using the patients’ body mass index (BMI) as a severity indicator for AN (as specified in Diagnostic and Statistical Manual of Mental Disorders, 5th Edition), we included only patients who suffered from “severe” AN (BMI = 15–15.99 kg/m^2^) or “extreme” AN (BMI < 15 kg/m^2^). In our clinical view, it would have been inappropriate if we had not offered neurosurgical treatment to these cases of severe, life-threatening AN. As argued in the context of DBS for treatment-refractory depression (7), in which a lower limit of 2-year illness duration was advocated as an inclusion criterion for DBS, we believe that disease severity and refractoriness are more important eligibility criteria than illness duration, at least in certain cases in psychiatric neurosurgery. Notwithstanding, we agree with Pugh et al. that our operational definitions of illness duration and treatment refractoriness were less strict than typically used and recommended. From a research perspective, the use of a less strict eligibility criterion could have constrained the external validity of our study. It is unlikely, however, that its use affected the internal validity and our conclusion on the potential clinical utility of capsulotomy in AN treatment.

In our study, the primary clinical outcome measure consisted of BMI, arguably the most important outcome criterion in AN, along with several well-established clinical rating scales for obsessive–compulsive symptoms, depression, anxiety, and social disability (1). Unfortunately, the clinical assessment did not include measures of the cognitive (e.g., impaired self-control, distorted perception of own body weight and shape) and behavioral aspects (e.g., binge-eating, purging) of AN, as pointed out by Pugh et al. The main reason to not include such measures was that, at the time of the surgery, there were no reliable and valid Chinese versions of clinical instruments available to evaluate these psychopathological aspects of AN. For example, the Chinese version of the Eating Attitudes Test has only recently been validated (8).

It is also important to underline that the patients who participated in our study could initially choose between stereotactic ablation and DBS. In contrast to the patient experiences reported by Pugh et al., most patients (more than 80% of all patients) who were eligible for neurosurgical treatment in our hospital hold relatively positive attitudes and perceptions about ablative surgery, favoring capsulotomy over DBS. The main reason given by patients for choosing capsulotomy was that this procedure had a lower risk of complications affecting their facial appearance than DBS. In our research report (1), we indicated that capsulotomy produced marked clinical benefits to the patients, but the intervention was also associated with several short-term side effects, including urinary incontinence (n = 7), sleep problems (n = 8), and fatigue (n = 6). Long-term side effects included behavioral disinhibition (n = 6), memory problems (n = 3), and lethargy (n = 4). Notwithstanding, the clinical improvements seen in the patients seem to us promising enough to warrant further research into this neurosurgical intervention for AN.

In our hospital, we provide both neurosurgical treatment options to carefully selected patients with severe and refractory AN because we believe that each intervention has its own advantages and disadvantages. From this perspective, as noted also by Zrinzo et al. (3), the commentary of Pugh et al. was not well balanced but biased toward the advantages of DBS. Yet, it should be realized that the clinical evidence on the efficacy of DBS for severe and refractory AN is actually quite limited. Since the first research report on DBS for AN (7), only a few case series have been published (**Table 1**).

**Table 1:** Studies of deep brain stimulation treatment for anorexia nervosa.

Reference	N	Gender	Age of surgery (years)	Target	Duration of FU (months)	BMI		Comorbidities (type, n, scores at baseline/LFU)
						Baseline	LFU	
McGregor et al. 2010	1	Female	52	Subgenual cingulate cortex	3	18.0	19.1	NA
Lipsman et al. 2017	16	Female	34 ± 8	Subcallosal cingulate	12	13.8 ± 1.5	17.3 ± 3.4	OCD: 16, 27.9 ± 8.6/14, 19.7 ± 10.3^a^ Anxiety: 16, 38.0 ± 15.6/14, 19.7 ± 18.4^b^
Blomstedt et al. 2017	1	Female	56	MFB in the posterior hypothalamic area	36	16.2	14.3	Anxiety: 1, 34/1, 15^c^ Depression:1, 22/1, 13^d^
Zhang et al. 2013	4	Female	17 ± 1	NAc	1	12.1 ± 0.8	15.7 ± 1.9	NA
Wang et al. 2013	2	Female	23 ± 5	NAc	12	13.1 ± 0.2	19.4 ± 1.4	OCD: 2, 22 ± 7/2, 14 ± 5^a^ Anxiety: 2, 29.5 ± 5.5/2, 7.5 ± 0.5^c^
Lipsman et al. 2013	6	Female	38 ± 11	Subcallosal cingulate	9	13.7 ± 1.4	16.6 ± 3.2	OCD: 6, 25.0 ± 10.9/6, 13.2 ± 6.9^a^ Anxiety: 6, 31.2 ± 18.8/6, 21.7 ± 14.1^b^ Depression: 6, 17.8 ± 8.2/6, 10.7 ± 8.4^d^
Wu et al. 2013	4	Female	17 ± 1	NAc	9-50	11.9 ± 1.2	19.6	OCD: 3, 20/3, 1.7^a^ Anxiety: 1, 19/1, 2^c^

N, number of patients; MFB, medial forebrain bundle; NAc, nucleus accumbens; BMI, body mass index; FU, follow-up; LFU, last follow-up.

a Yale–Brown Obsessive Compulsive Scale (Y-BOCS).

b Beck Anxiety Inventory (BAI).

c Hamilton Rating Scale for Anxiety (HAM-A).

d Hamilton Rating Scale for Depression (HAM-D).

The largest case series involved only 16 patients who were followed up for 1 year (6). In general, these studies suggest that the clinical efficacy of DBS for severe and refractory AN is, as yet, not satisfactory. For example, in the aforementioned DBS study of 16 patients with AN (6), 5 out of 14 patients (36%) (2 patients were lost to follow-up) still had their BMI under 16 at the last follow-up. (**Table 1**). Accordingly, we consider both ablative surgery and DBS as promising experimental therapeutic interventions that justify clinical use and further research. As emphasized by Zrinzo et al. (3), we believe that a fully informed patient who is capable of giving voluntary consent should make his or her own decision about whether or not to utilize an experimental treatment that is provided by medical professionals. Similarly, restricting investigational neurosurgical treatments for AN to DBS limits the breadth of psychiatric surgery research, as well as limiting the choices of individual patients.

## Author Contributions

CZ, WL, DL, GL, and BS were all involved in the conceptual design of the paper. WX developed the table, and ZL and CZ developed the initial draft from intellectual comments from all authors.

## Conflict of Interest Statement

BS received research support from SceneRay and PINS (donated devices); DL and CZ have received honoraria and travel expenses from the Deep Brain Stimulation industry (Medtronic, PINS, SceneRay). The remaining authors declare that the research was conducted in the absence of any commercial or financial relationships that could be construed as a potential conflict of interest.
